# Supplementary material for: Heterosis and Differential DNA Methylation in Soybean Hybrids and Their Parental Lines
Source: Plants (Basel). 2022 Apr 22;11(9):1136. doi: 10.3390/plants11091136 (PMC9102035; doi:10.3390/plants11091136)
Supplement: Supplementary file 1 [file plants-11-01136-s001.zip › Supplementary Figure.pdf]

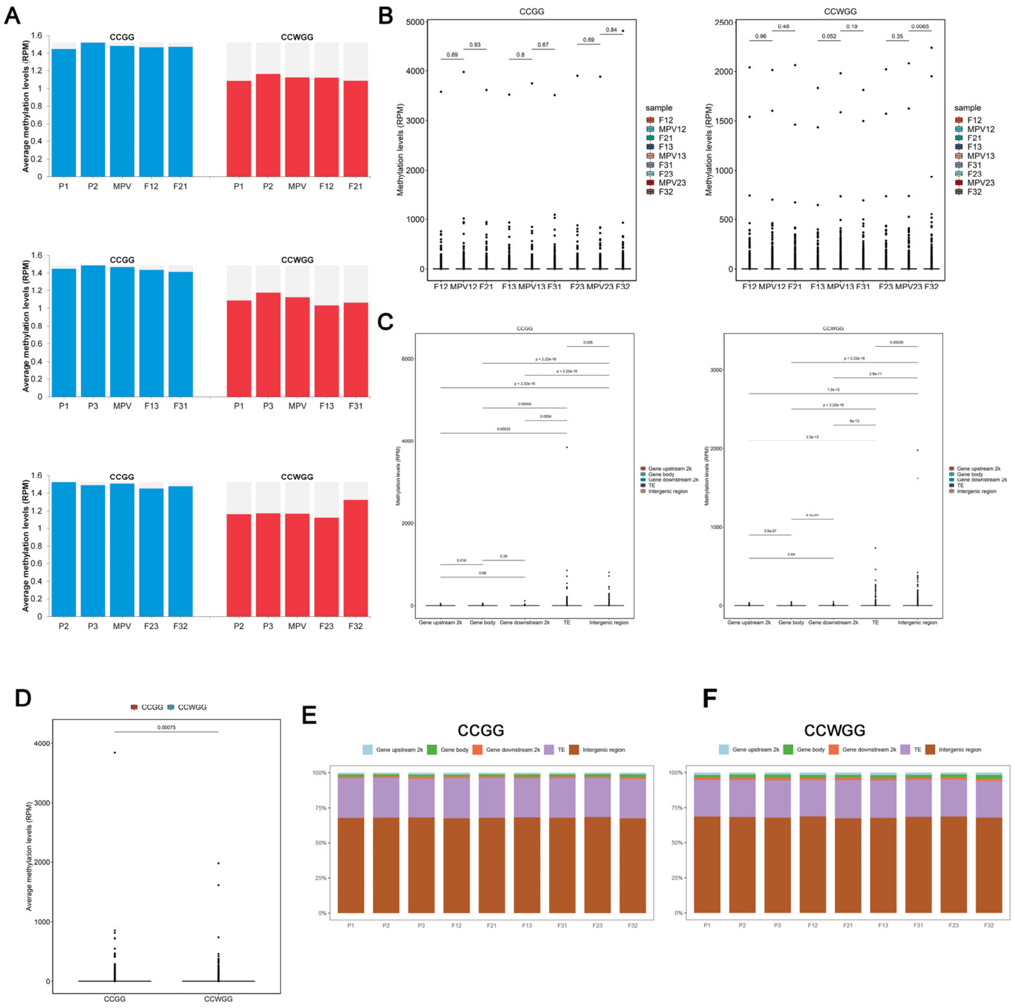

Figure S1. DNA methylation patterns of three combinations in soybean contemporary seeds. (A) and (B) Relative average methylation levels in different genomic elements. (C) Distribution of methylation sites in soybean genome. (D) The significant difference of CCGG and CCWGG methylation levels. The number above black line of two sites is *p* value determined by the paired Student's t-test (E) and (F) Distribution of super-methylated sites in genome.

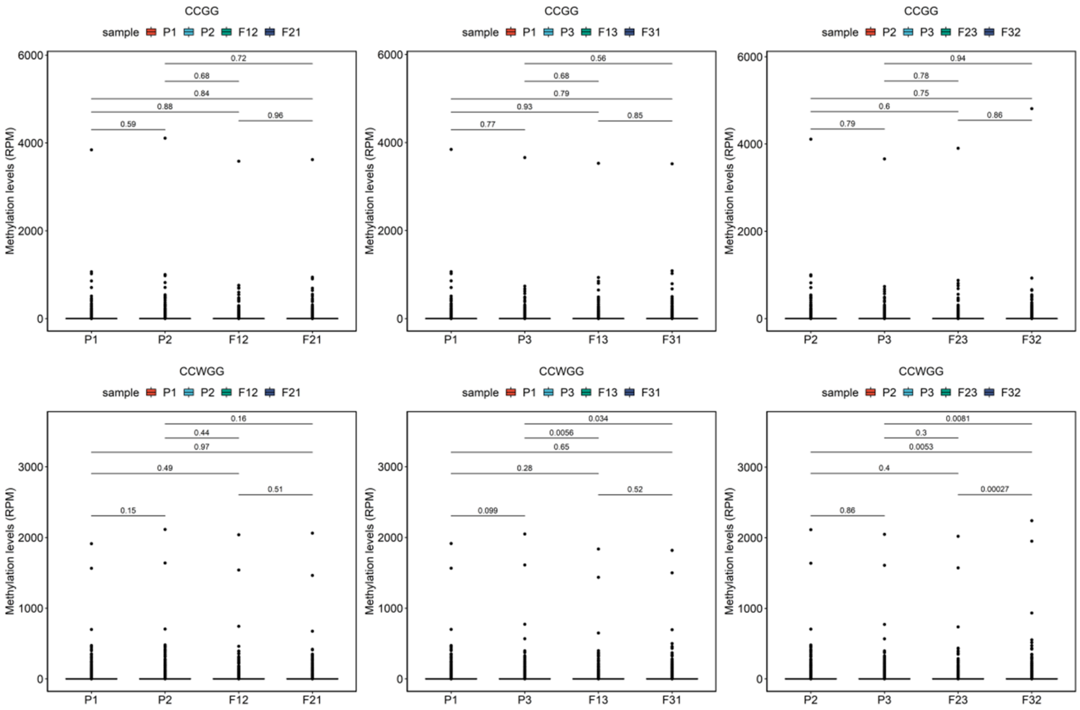

Figure S2. Comparison of average methylation level of any two samples among three combinations for CCGG and CCWGG sites.

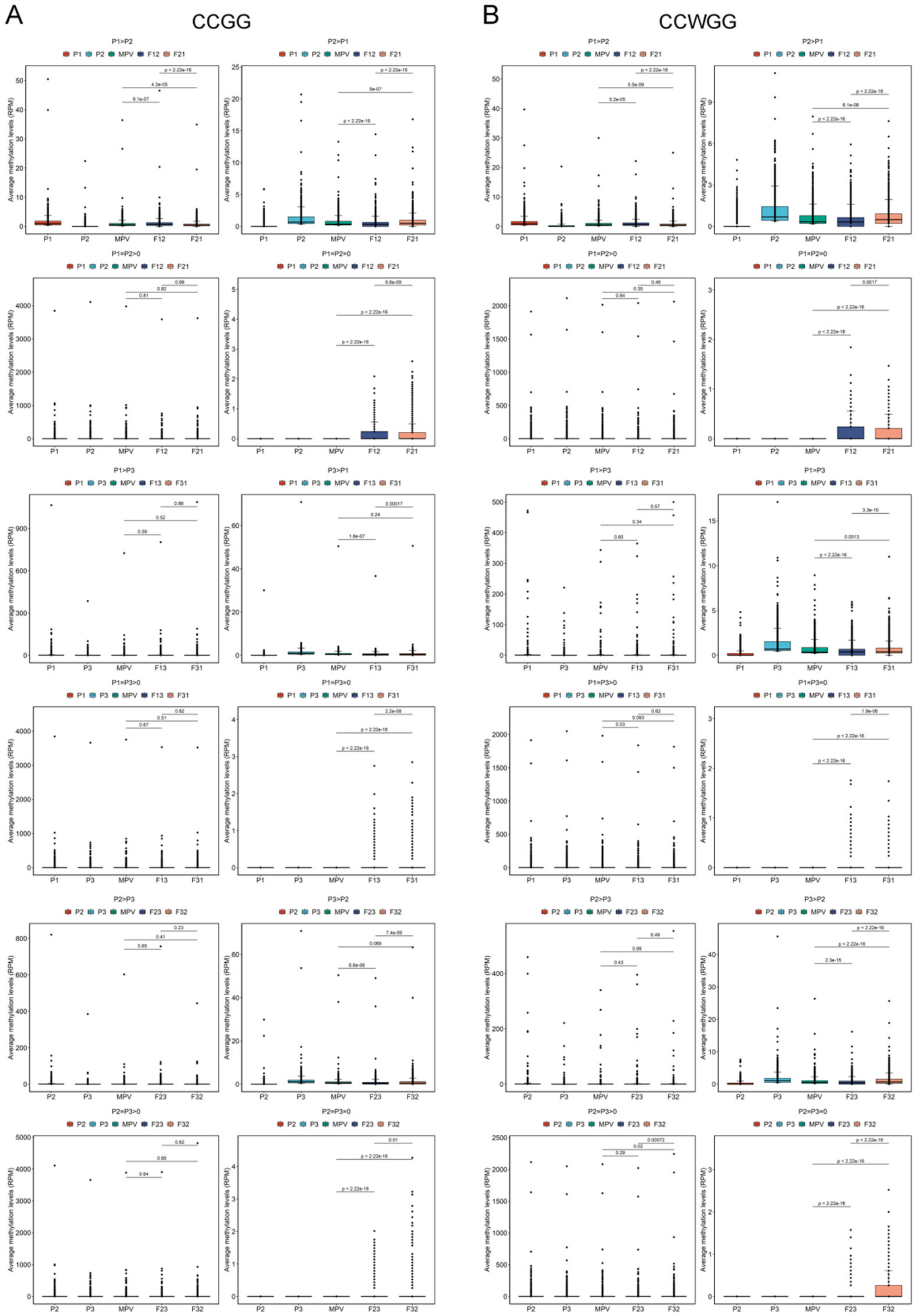

Figure S3. Association of CCGG (A) and CCWGG (B) context DNA methylation in hybrids with parental methylation status.

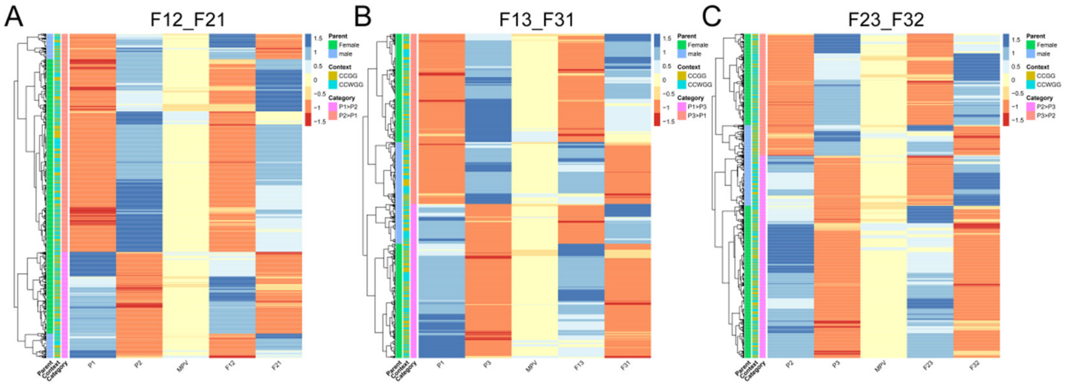

Figure S4. The methylation levels in parental selective difference sites for all combinations. (A) to (C) F12_F21/F13_F31/F23_F32, the parental selective difference sites of reciprocal hybrids, e.g. F12_F21 means these unequal sites between F12 and F21.
