# Supplementary figures and images for: Heterosis and Differential DNA Methylation in Soybean Hybrids and Their Parental Lines
Source: Plants (Basel). 2022 Apr 22;11(9):1136. doi: 10.3390/plants11091136 (PMC9102035; doi:10.3390/plants11091136)

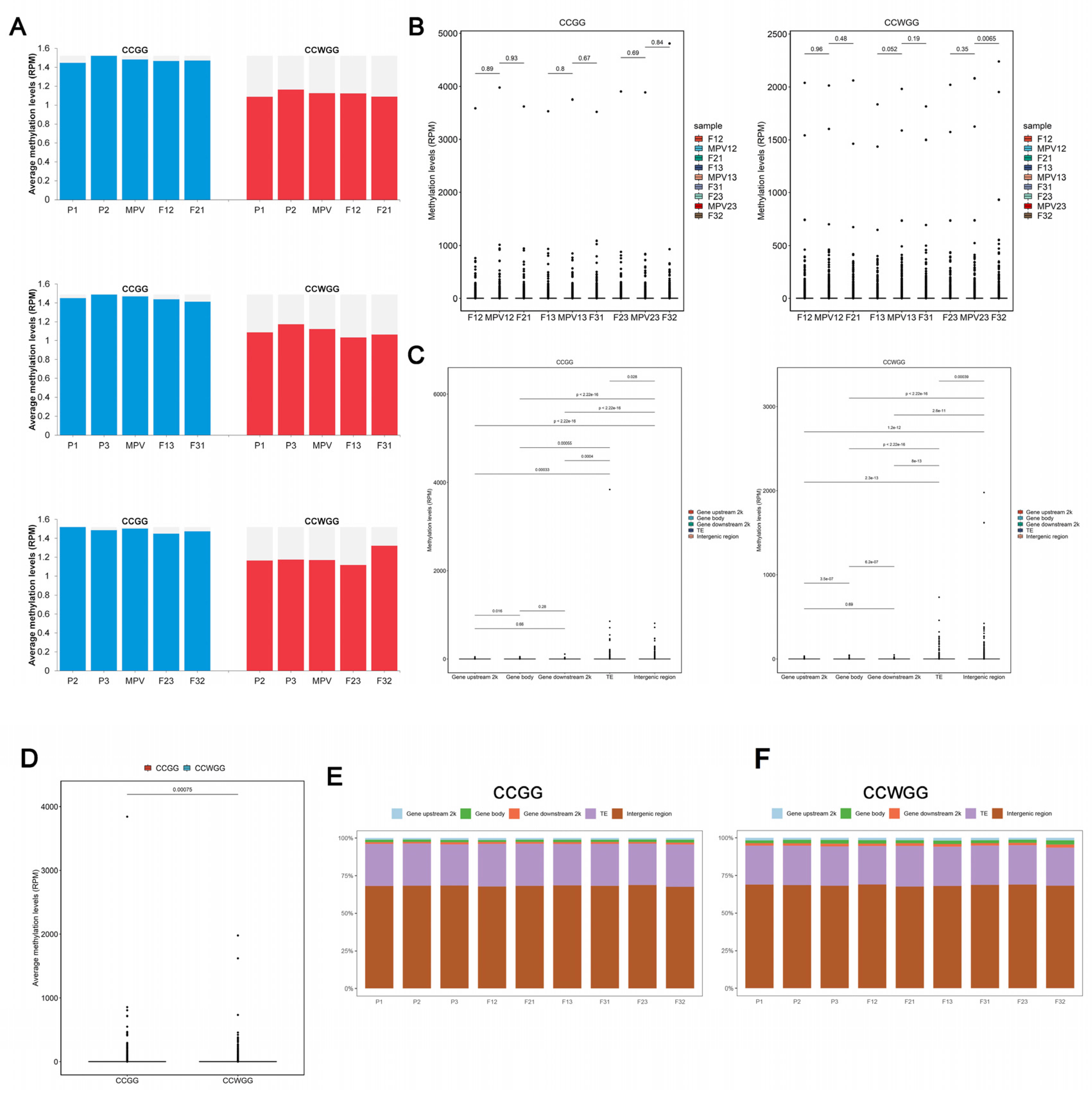

Supplement: Supplementary file 1 [file plants-11-01136-s001.zip › Figure S1.jpg]

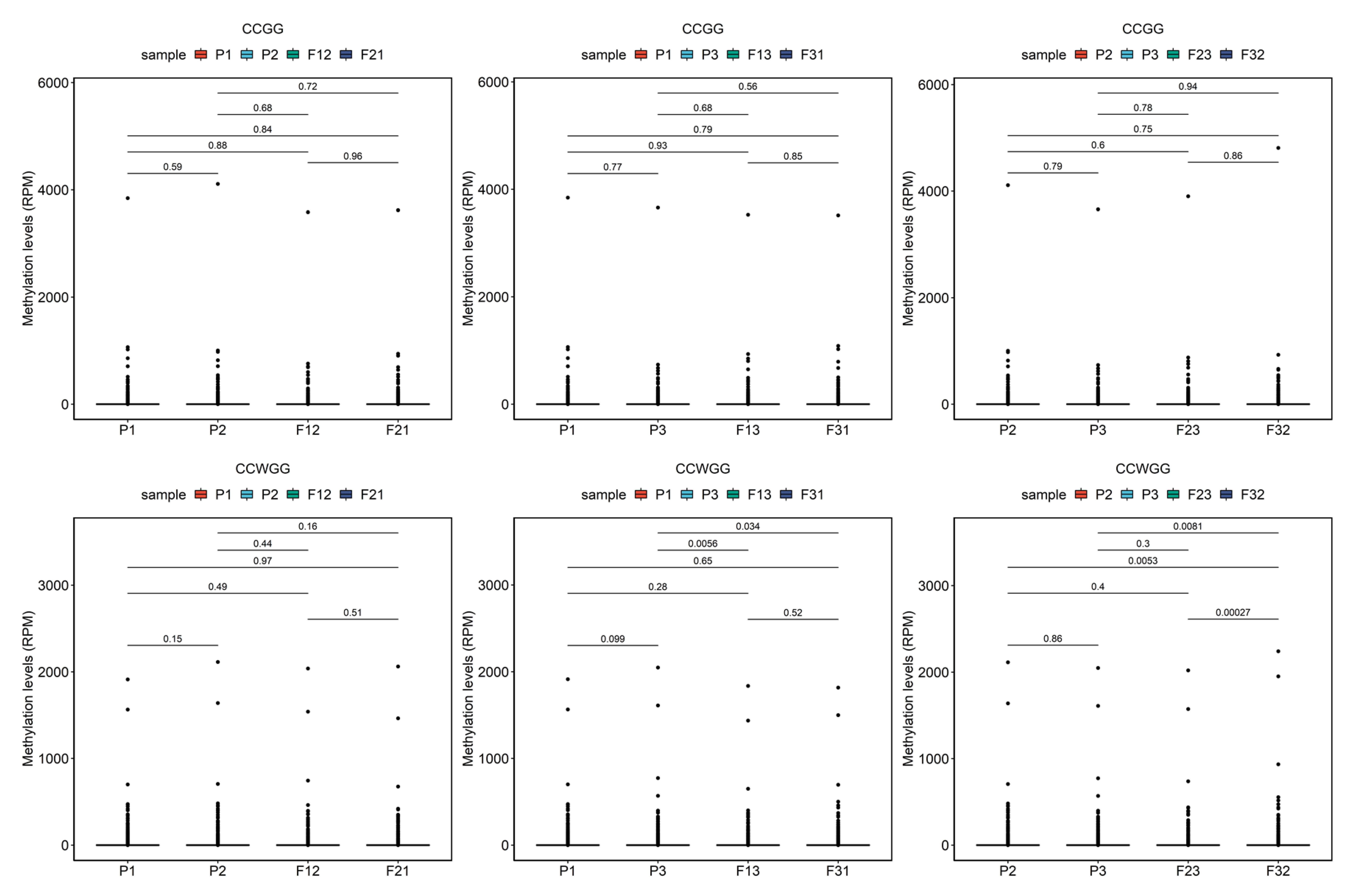

Supplement: Supplementary file 1 [file plants-11-01136-s001.zip › Figure S2.jpg]

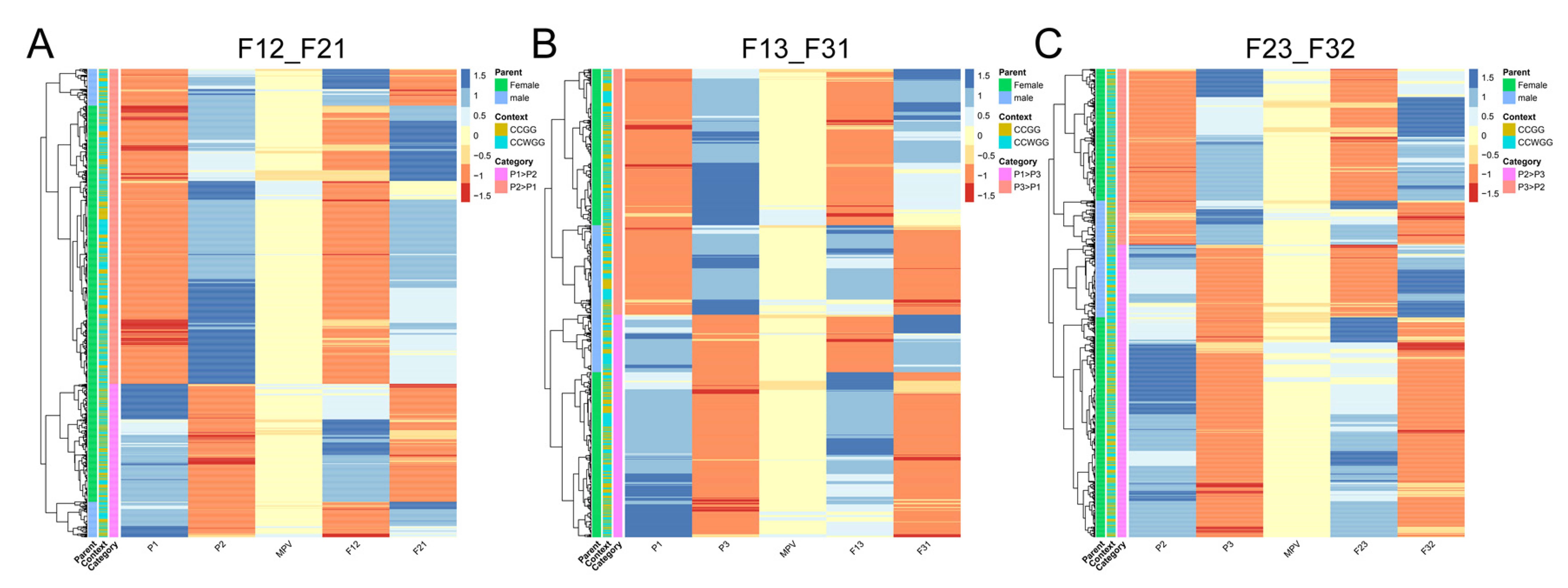

Supplement: Supplementary file 1 [file plants-11-01136-s001.zip › Figure S4.jpg]
